# Improving Systematic Review Updates With Natural Language Processing Through Abstract Component Classification and Selection: Algorithm Development and Validation

**DOI:** 10.2196/65371

**Published:** 2025-03-27

**Authors:** Tatsuki Hasegawa, Hayato Kizaki, Keisho Ikegami, Shungo Imai, Yuki Yanagisawa, Shuntaro Yada, Eiji Aramaki, Satoko Hori

**Affiliations:** 1 Division of Drug Informatics Keio University Faculty of Pharmacy Tokyo Japan; 2 Nara Institute of Science and Technology Nara Japan; 3 Faculty of Library, Information and Media Science University of Tsukuba Tsukuba Japan

**Keywords:** systematic review, natural language processing, guideline updates, bidirectional encoder representations from transformer, screening model, literature, efficiency, updating systematic reviews, language model

## Abstract

**Background:**

A challenge in updating systematic reviews is the workload in screening the articles. Many screening models using natural language processing technology have been implemented to scrutinize articles based on titles and abstracts. While these approaches show promise, traditional models typically treat abstracts as uniform text. We hypothesize that selective training on specific abstract components could enhance model performance for systematic review screening.

**Objective:**

We evaluated the efficacy of a novel screening model that selects specific components from abstracts to improve performance and developed an automatic systematic review update model using an abstract component classifier to categorize abstracts based on their components.

**Methods:**

A screening model was created based on the included and excluded articles in the existing systematic review and used as the scheme for the automatic update of the systematic review. A prior publication was selected for the systematic review, and articles included or excluded in the articles screening process were used as training data. The titles and abstracts were classified into 5 categories (Title, Introduction, Methods, Results, and Conclusion). Thirty-one component-composition datasets were created by combining 5 component datasets. We implemented 31 screening models using the component-composition datasets and compared their performances. Comparisons were conducted using 3 pretrained models: Bidirectional Encoder Representations from Transformer (BERT), BioLinkBERT, and BioM- Efficiently Learning an Encoder that Classifies Token Replacements Accurately (ELECTRA). Moreover, to automate the component selection of abstracts, we developed the Abstract Component Classifier Model and created component datasets using this classifier model classification. Using the component datasets classified using the Abstract Component Classifier Model, we created 10 component-composition datasets used by the top 10 screening models with the highest performance when implementing screening models using the component datasets that were classified manually. Ten screening models were implemented using these datasets, and their performances were compared with those of models developed using manually classified component-composition datasets. The primary evaluation metric was the F10-Score weighted by the recall.

**Results:**

A total of 256 included articles and 1261 excluded articles were extracted from the selected systematic review. In the screening models implemented using manually classified datasets, the performance of some surpassed that of models trained on all components (BERT: 9 models, BioLinkBERT: 6 models, and BioM-ELECTRA: 21 models). In models implemented using datasets classified by the Abstract Component Classifier Model, the performances of some models (BERT: 7 models and BioM-ELECTRA: 9 models) surpassed that of the models trained on all components. These models achieved an 88.6% reduction in manual screening workload while maintaining high recall (0.93).

**Conclusions:**

Component selection from the title and abstract can improve the performance of screening models and substantially reduce the manual screening workload in systematic review updates. Future research should focus on validating this approach across different systematic review domains.

## Introduction

Systematic reviews are based on evidence-based medicine and constitute crucial sources of information for health care professionals and policy makers to access the latest information in their fields and thereby aid decision-making [1,2]. Systematic reviews of randomized controlled trials are highly valued and frequently referenced in the development of clinical practice guidelines by authoritative organizations, such as the World Health Organization (WHO), representing a crucial foundation for evidence-based health care decision-making. When systematic reviews are outdated or fail to encompass all the available evidence, they risk misinforming decision makers and other stakeholders [3]. Therefore, systematic reviews should be regularly updated. Shojania et al [4] analyzed 100 quantitative systematic reviews that were registered with the ACP Journal Club and reported a median survival time of 5.5 years for systematic reviews [4]. However, most systematic reviews remain outdated, and Hoffmeyer et al [2] reported that 88% of reviews in the Cochrane Database of Systematic Reviews had not been updated for 5.5 years.

A significant challenge in updating systematic reviews is the workload associated with article screening that, for systematic review research and updates, is usually performed using the following 5 steps [5,6]: (1) identifying databases relevant to the topic, (2) finding potentially relevant articles through a tailored search strategy, (3) screening articles based on titles and abstracts, (4) selecting articles for detailed analysis from the screened texts, and (5) extracting and synthesizing data from the selected articles. The significant effort required for these steps as well as the stringent requirement that these tasks be performed independently by 2 or more individuals [7], seems to contribute to the low rate of systematic review updates.

Recent advancements in natural language processing (NLP) technology have led to numerous attempts to automate these screening tasks. Qin et al [8] used an ensemble learning model that integrated multiple Bidirectional Encoder Representations from Transformer (BERT) models that were trained on the titles or abstracts and results of articles screened before an update of a systematic review on the treatment of type 2 diabetes with sodium-glucose cotransporter-2 inhibitors. Their model achieved a sensitivity of 96%, a specificity of 78%, and a 64.1% reduction in workload. While these models, like any NLP applications, require careful consideration of potential challenges such as overfitting and validation across different domains, these promising, along with other studies, have established the viability of NLP-based screening automation.

Building upon these achievements, we explored a novel approach focusing on how models process and learn from abstract information. Scientific abstracts contain distinct components (eg, introduction, methods, results, and conclusions) that serve different rhetorical functions and potentially contribute differently to determining article relevance for systematic reviews. While current approaches typically treat abstracts as uniform text, we hypothesized that selective training on specific abstract components might help address common challenges in NLP-based screening models, including the risk of overfitting large volumes of text data. By focusing on the most relevant information for the systematic review’s inclusion criteria, this approach could potentially enhance model performance compared to traditional methods that process entire abstracts as uniform text.

This component-based approach potentially offers several advantages. For example, methods sections often contain crucial information about study design and participant characteristics, while results sections present outcome measures—both critical factors when selecting articles for inclusion in systematic reviews. By selectively focusing on these key components, models might learn more effectively from smaller, more targeted training data.

Scientific journals have widely adopted structured abstracts with clearly labeled components (eg, Introduction, Methods, Results, and Conclusion) since the early 1990s [9]. However, systematic reviews often need to process both structured and unstructured abstracts. To implement our component-based screening approach effectively across all articles, we needed to develop an NLP-based automatic abstract component classifier that could systematically identify and process abstract components regardless of their original format (structured or unstructured).

In this study, we demonstrate the usefulness of a screening model that learns by selecting abstract components as a novel method for improving the performance of screening models. In addition, we construct a screening model with an NLP-based automatic abstract classifier that classifies abstracts by component.

## Methods

### Overview

**Figure 1:**
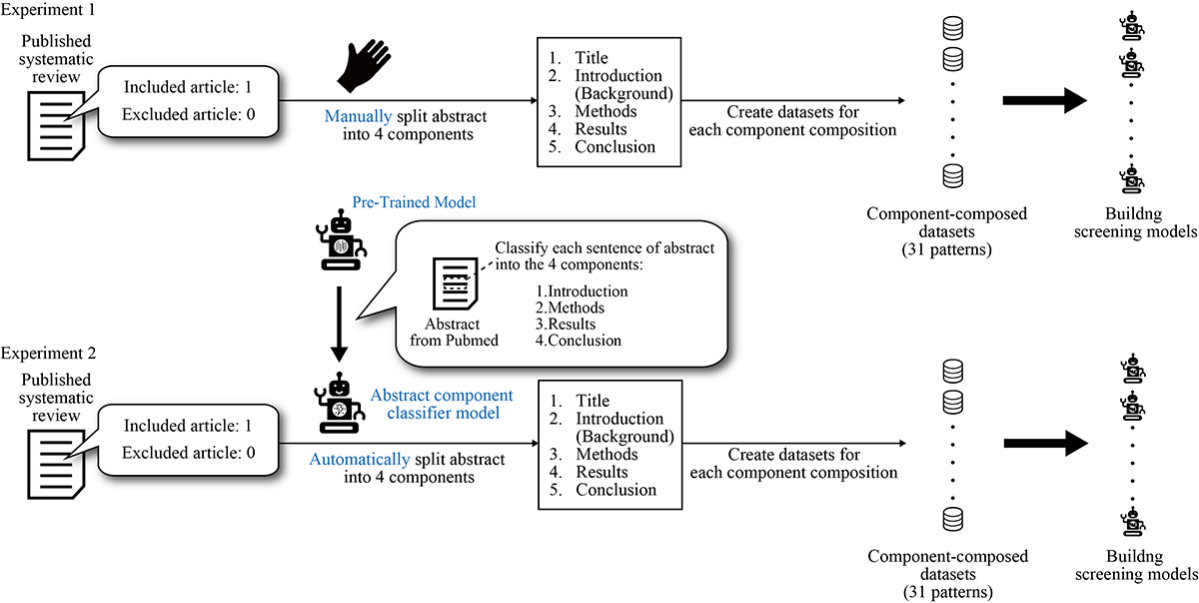
Overview of this study experiments.

Our study consisted of 2 experiments (Figure 1). First, to evaluate the impact of selecting titles and abstract components of articles on the development of the screening model, we constructed datasets with different combinations of titles and 4 abstract components and built a screening model using these datasets (under subheading Experiment 1). Second, to automate the process from dataset creation to classifier development, we developed a model for classifying abstract components of articles (Abstract Component Classifier Models) and built a screening model using the abstract components identified by the Abstract Component Classifier Model (under subheading Experiment 2).

For identifying both included and excluded articles, our study followed a systematic process: first, articles were identified and curated based on our selection criteria, then their abstracts were systematically subdivided into components for analysis. This component classification process was performed manually in Experiment 1 and automatically using our classifier in Experiment 2. The details of each step are described in the following sections.

### Experiment 1: Evaluation of the Impact of Selecting Titles and Abstract Components of Articles on the Development of the Screening Model

#### Data Sources

We scrutinized the study “Adverse events in people taking macrolide antibiotics versus placebo for any indication,” published by Hansen et al [10] in the Cochrane Database of Systematic Reviews on January 18, 2019 as the data source. The data source was a systematic review that assessed adverse events in randomized controlled trials of patients treated with antibiotics compared with a placebo. This review was selected for 2 reasons. First, the number of studies included in the analysis was substantial, with 184 unique studies (reported across 314 publications), providing sufficient data for implementing the screening model. Second, the systematic review included randomized controlled trials, consistent with other systematic reviews analyzing treatment effects.

**Figure 2:**
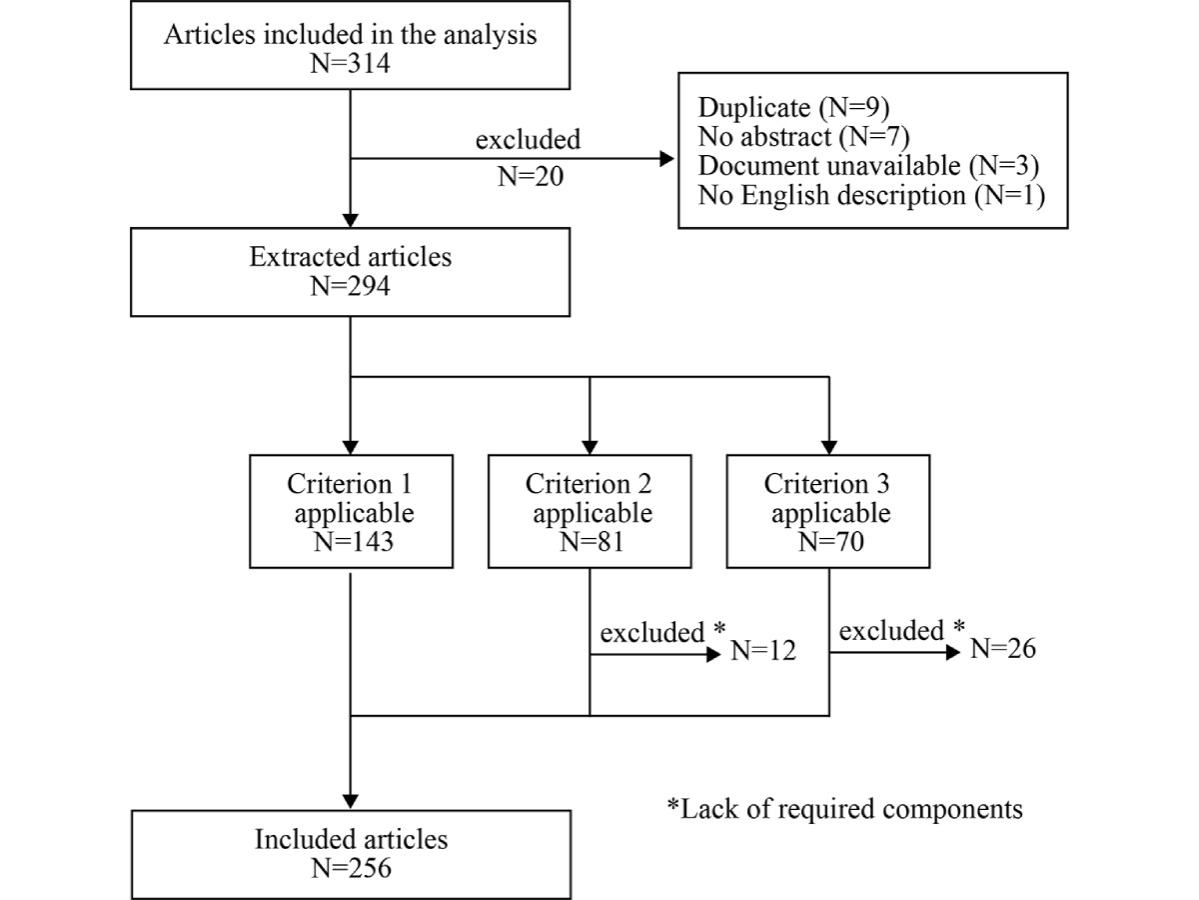
Screening process for included articles in this study.

To identify the included articles, we analyzed the 184 studies (314 articles) in the inclusion list for the systematic review. The screening process for included articles is shown in Figure 2. We selected articles that met any of the criteria with both the title and abstract written in English, the criteria are mentioned in Textbox 1.

**Inclusion criteria for included articles.**

Criterion 1:

- The abstract was structured into 4 components, introduction (background), methods, results, and conclusion, or equivalent, from which the title and the aforementioned 4 components could be extracted.

Criterion 2:

- The abstract was not divided into exactly four components but followed a structured format (eg, 3 or 5 components) and could be reclassified by researchers into 4 components—introduction (background), methods, results, and conclusion, or equivalent—from which the title and the aforementioned four components could be extracted.

Criterion 3:

- The abstract did not follow a structured abstract, but the text could be classified into four components—introduction (background), methods, results, and conclusion, or equivalent—from which the title and the aforementioned four components could be extracted.

The criteria for the 4 components equivalent to introduction (background), methods, results, and conclusion, as well as for excluding text, are shown in Table S1 in Multimedia Appendix 1. For the classification of criteria 2 and 3, two researchers (TH and YY) independently classified each sentence in the abstract into 4 components. Disagreements, if any, were resolved through discussion. In our study, all abstracts were assumed to be written in the following order: introduction (background), methods, results, and conclusion. Therefore, according to the criteria listed in Table S1 in Multimedia Appendix 1, if the sentences before and after a sentence classified as results were related to methods, the sentence was classified as methods. In total, 256 articles were included.

To identify the excluded articles, we did not contact the original review authors but instead searched MEDLINE (Ovid) and the Cochrane Central Register of Controlled Trials using the same search terms and search periods as those used in the systematic review. The search terms and formulas used are listed in Table S2 of Multimedia Appendix 1. Articles that met the above criteria 1 or 2 with both the title and abstract written in English were then extracted, and the “included articles” in our study and any articles similar to those were excluded. The following exclusion criteria were applied: (1) articles without English abstracts, as they would not be suitable for our model training, (2) articles without abstracts, particularly older publications or those with only titles, and (3) articles with unstructured abstracts lacking clear background, methods, results, and conclusion sections, as they would not be appropriate for our component classification task. We did not include articles that only met criterion 3 (articles with title and abstract written in English but not meeting criterion 1 or 2) as negative instances, as we had already obtained a sufficient number of excluded articles from articles meeting criteria 1 and 2. The classification of the 4 components was conducted with reference to the criteria listed in Table S1 in Multimedia Appendix 1. For the classification of criterion 2, we evaluated the agreement rate between 2 researchers. The simple agreement rate was 98.76% (5752/5824; 95% CI 98.47%-99.05%). It was considered sufficiently high; therefore, one researcher (TH) performed the classification.

In addition, the decision regarding duplicates in the “included articles” in our study was made using the SequenceMatcher object from the difflib library. The similarity ratio of the text of each component in the excluded articles to the text of the same components in all the “included articles” and extracted the highest value was calculated. Articles with a combined similarity ratio value of 4.0 or more, or with a similarity ratio of the title of more than 0.8, were excluded. Through manual verification of the flagged cases, we confirmed 1 complete text duplication and 2 cases of substantial text similarity above our thresholds. These 3 cases were excluded from the analysis, confirming the appropriateness of our automated duplicate detection criteria. A similar assessment using the title was also performed for the studies excluded from the article dataset. Duplicates among the excluded articles were not excluded because duplicates might exist in the articles to be scrutinized for practical use. Ultimately, 1261 articles were extracted.

We created 31 different component-composed datasets from 5 components in the extracted articles. These included datasets containing only the title, introduction, methods, results, or conclusions; datasets containing all 5 components; and datasets containing any combination of 2, 3, or 4 of these components. All variations are shown in Table S3 in Multimedia Appendix 1.

#### Development of Screening Models

From each component-composed dataset, we created training, validation, and test sets. Included and excluded articles were treated as positive and negative data, respectively. For positive data, 40 articles were randomly extracted as the test set, and the remaining 216 articles were randomly divided into training and validation sets in a 9:1 ratio. Negative data were randomly assigned to the training and validation sets to ensure 1:1 positive-to-negative data. In the test set, 960 articles were assigned randomly. For articles exceeding the maximum sequence length of 512 tokens, the text was truncated during the model training phase. All shorter inputs were padded to ensure uniform input length. This means that for articles exceeding the token limit, information from the latter portions of the text was not used during model training.

We developed screening models that predicted binary labels for the test set using the transformer library. During model development, different learning rates (Lr; 6e-7, 2e-6, and 6e-6) were evaluated using the validation set. Screening models were developed using 3 pretrained language models: Bidirectional Encoder Representations from Transformers (BERT; bert-large-uncased [11]), BioLinkBERT (BioLinkBERT-Large) [12], and BioM- Efficiently Learning an Encoder that Classifies Token Replacements Accurately (ELECTRA) (BioM-ELECTRA-Large-SQuAD2) [13]. BERT, introduced by Google in 2018, is pretrained on 2.5 billion words from the English Wikipedia and 800 million words from BookCorpus [14]. It uses a Transformer Encoder using a method known as attention, which can effectively incorporate information from distant positions within a text.

We used this as the baseline pretrained model. BioLinkBERT is a pretrained model developed by Yasunaga et al [15] at Stanford University. Traditional BERT models cannot learn from the text of hyperlinks and citation links used in pretraining documents. The base model, LinkBERT, can capture these document links. BioLinkBERT [12] was pretrained on 3.1 billion words from PubMed abstracts and documents from their hyperlinks and citation links. BioM-ELECTRA-Large-SQuAD2 is a model developed by Alrowili and Shanker [16] from the University of Delaware that trains BioM-ELECTRA-Large on the SQuAD2.0 dataset to enhance question-answering capabilities. BioM-ELECTRA is based on the ELECTRA model, introduced by Clark et al [17] in 2020, which improves the pretraining methods of BERT to achieve comparable or superior performance with fewer computational resources. BioM-ELECTRA was pretrained on 29,000 domain-specific PubMed abstracts, following the same domain-specific approach as PubMedBERT [16]. Among the pretrained models implemented in this study, BioLinkBERT and BioM-ELECTRA were the highest-performing models in the BLURB benchmark [18], which evaluates the performance of pretrained models in the biomedical literature.

#### Task and Metrics

For each pretrained model, we fine-tuned 31 screening models corresponding to all possible combinations of the 5 components (Title, Introduction, Methods, Results, and Conclusion) and compared their performances. In systematic review screening, minimizing false negatives (missed relevant articles) is critically important, as failing to include relevant articles could significantly impact review conclusions. While Recall would be an appropriate metric for this purpose, using it alone would favor models that simply classify all articles as positive, achieving perfect recall but poor practical utility.

To address this, we designated the Fβ-score as our primary evaluation metric. This score is the harmonic mean of recall and precision, where β determines their relative importance. During our preliminary analysis, we tested several values of β to determine the optimal balance between recall and precision for our specific use case. Compared to other potential metrics, such as maximizing specificity at fixed recall thresholds, the Fβ-score provides a single, interpretable measure that enables explicit control over the balance between false positives and false negatives. We set β=10 to strongly prioritize recall while maintaining some consideration for precision, reflecting the practical trade-off in systematic review screening where missing relevant studies is more problematic than including irrelevant ones for subsequent manual screening. This value was chosen based on both empirical testing and principles of systematic review methodology where comprehensiveness in literature identification is critical. While this strong prioritization through β=10 results in increased false positives, this trade-off is considered acceptable in the context of systematic reviews, where missing relevant studies could significantly impact review conclusions. This approach also ensures that a model simply classifying everything as positive would be penalized through its low precision component, despite achieving perfect recall.

We calculated the average F10-Score evaluated 5 times using 5 datasets with different assignments for each component composition and compared these averages between the screening models. To elaborate, for each of the 31 possible component combinations, we created 5 distinct datasets using different random samples of included and excluded articles for training, validation, and test sets. This process of splitting the data into training, validation, and test sets was repeated 5 times, ensuring that each of the 5 datasets was distinct, and allowing us to evaluate the model performance with more reliability and less influence from any specific split. Specifically, from the 256 included articles, we randomly selected 40 articles to form the test set. The remaining 216 articles were randomly divided into training and validation sets using a 9:1 ratio. The excluded articles were assigned randomly to the training and validation sets, and 960 were randomly assigned to the test set. By averaging the F10-scores across these 5 datasets, we aimed to evaluate model performance across multiple splits, providing more stable evaluation metrics by mitigating variations due to randomness in data sampling.

The number of epochs for training the screening models was set to 32, patience for early stopping to 3, and weight decay to 0.01. In addition, we compared the screening model with the highest average F10-Score with those of other component compositions among the 3 Lr: 6e-6, 2e-6, and 6e-7.

### Experiment 2: Development of a Screening Model Using Article Components as Determined by the Abstract Component Classifier Model

In Experiment 2, we aimed to construct a screening model by automating the division of abstract components, which was done manually in Experiment 1. To achieve this, we first developed an Abstract Component Classifier Model to automate the partitioning of abstract components.

#### Development of the Abstract Component Classifier Model

We obtained 25,584 articles with abstracts registered in PubMed from May 2 to 8, 2018, and from August 16 to 19, 2022, using the “fha[Filter]” search term, which is a PubMed filter tag that helps identify articles with structured abstracts. From the article base, we extracted 5331 articles that met the criteria of having abstracts structured into 4 components (introduction or background, methods, results, and conclusion) with both the title and abstract written in English, allowing the extraction of these components along with the title. Each component was classified according to the criteria listed in Table S1 in Multimedia Appendix 1. Using the tokenized object from the Natural Language Toolkit library for initial sentence splitting, we divided the abstracts into individual sentences. Each sentence was then manually labeled with exactly one mutually exclusive component tag “Introduction [I], Methods [M], Result [R], Conclusion [C]”. Ultimately, we extracted 62,798 sentences, comprising 11,961; 17,154; 23,204; and 10,479 sentences for the introduction, methods, results, and conclusion, respectively (PubMed dataset). In addition, abstracts of the articles extracted from the systematic review in Experiment 1 were divided into sentences and labeled (Systematic Review Dataset). The dataset included 3477, 6265, 7091, and 3038 sentences for the introduction, methods, results, and conclusion, respectively, with a total of 19,871 sentences.

From the labeled sentences of the PubMed Dataset, we created training, validation, and test sets. We randomly allocated 4000 sentences (8:1:1) to the training, validation, and test sets. These datasets comprised equal proportions of samples for each label to prevent performance deterioration owing to unbalanced samples.

Abstract Component Classifier models were implemented to predict the sentence labels using the transformer library. We developed them from 4 pretrained models: (BERT; bert-large-uncased [11]), BioLinkBERT (BioLinkBERT-Large) [12], 2 types of BioM-ELECTRA, BioM-ELECTRA-Large-SQuAD2 [13] and BioM-ELECTRA-Large-Discriminator [19]. BioM-ELECTRA-Large-Discriminator is a model that, unlike the BioM-ELECTRA-Large-SQuAD2, does not undergo fine-tuning with SQuAD2; it uses the same pretraining corpus as BioM-ELECTRA-Large-SQuAD2.

We trained the classifier models on the training and validation sets and evaluated the model performance using the test set. For the Abstract Component Classifier Model, we limited our parameter tuning to Lr, as our primary objective was to confirm the basic capability of the component classification model rather than maximizing performance through extensive parameter optimization. This focused approach was supported by the strong baseline performance achieved with basic parameter settings.

Then, we classified the sentences in the PubMed dataset and evaluated their performance. The primary evaluation metric was the macro–*F*_1_-score (*F*_1_-score). The number of epochs for training the screening models was set to 32, patience for early stopping to 3, and weight decay to 0.01.

Similarly, we conducted the same analysis for the Systematic Review Dataset, and the model with the highest *F*_1_-score was also selected as the Abstract Component Classifier Model, which was used for subsequent analysis.

#### Development of a Screening Model Using Article Components as Determined by the Abstract Component Classifier Model

As described in Experiment 1, we developed screening models using the same data sources and methods. However, we used the Abstract Component Classifier Model, which we developed to automatically classify abstract components of articles that were previously classified manually in Experiment 1. These abstracts were reclassified into 4 components based on the results of classification using the Abstract Component Classifier Model. If no sentence was allocated to any of the components in the classifier model, the component was treated as a document with 0 words. From the title data and reclassified component data, we created component-composed datasets. To focus on the most promising approaches while maintaining computational efficiency, we selected 10 datasets corresponding to the top 10 performing models from Experiment 1. This focused approach was selected to specifically examine whether the advantages observed with manual classification could be maintained when transitioning to an automated system; a key consideration for practical implementation. This selection allowed us to evaluate how well these high-performing component combinations performed when using automated classification instead of manual classification. Table S3 in Multimedia Appendix 1 lists the component composition used in the screening models, which were implemented using training datasets created from the selected 10 component-composed datasets. The primary endpoint was the F10-score, which was compared to the performance of 10 classifiers with the same component composition constructed in Experiment 1.

### Ethical Considerations

All analyses were conducted using previously published studies, and therefore, ethics approval and patient consent were unnecessary. This study does not include human subject information, primary data collection, or any form of experimentation involving individuals.

## Results

### Overview

All results reported in this section were obtained using the test set, which was completely separate from the training and validation sets used for model development.

#### Experiment 1: Evaluation of the Impact of Selecting Article Titles and Abstract Components on the Development of the Screening Model

##### Characteristics of the Number of Words in the Extracted Articles

The word count of the 1517 articles had a median of 276 (range 103-1099) words. After tokenization, 50 articles (including 2 included articles) exceeded the maximum sequence length of 512 tokens that our pretrained models could process in one iteration.

##### Performance of Screening Models

A detailed description of all datasets, including class proportions, is provided in Table S4 in Multimedia Appendix 1. Details of the final evaluation results on the held-out test set for the screening models with different Lr (6e-7, 2e-6, and 6e-6) are shown in Table S5-S7 in Multimedia Appendix 1. For the screening model using BERT (Table S4 in Multimedia Appendix 1), the model trained on the 4 components “Title + Methods + Results + Conclusion” demonstrated the highest average F10-Score (precision=0.35; recall=0.93; F10-Score=0.91). Conversely, the model with the lowest F10-Score was trained solely on the conclusion (precision=0.48; recall=0.77; F10-Score=0.75). The model trained on all 5 components ranked tenth in the average F10-Score of all 31 screening models. For BioLinkBERT (Table S5 in Multimedia Appendix 1), similar to BERT, the model trained on “Title + Methods + Results + Conclusion” recorded the highest F10-Score (precision=0.28; recall=0.95; F10-Score=0.93). The lowest F10-Score, as with BERT, was observed in the model trained only on the Conclusion (precision=0.58, recall=0.77; F10-Score=0.76). The model trained on all 5 components had the seventh highest average F10-Score of all 31 screening models. For the BioM-ELECTRA (Table S6 in Multimedia Appendix 1), the model trained on Methods + Conclusion achieved the highest F10-Score (precision=0.37; recall=0.89; F10-Score=0.88). The lowest F10-Score was obtained for the model trained on Title + Introduction + Methods (precision=0.32; recall=0.74; F10-Score=0.73). The model trained on all 5 components ranked 20 seconds in the average F10-Score among all the 31 screening models.

#### Experiment 2: Development of a Screening Model Using Article Components as Determined by the Abstract Component Classifier Model

##### Development of the Abstract Component Classifier Model

Table 1 lists the average results of 5 evaluations of the classifier models using the PubMed Dataset. The main evaluation metric, the *F*_1_-score was 0.89 for BERT; Lr:6e-6, 0.92 for BioLinkBERT; Lr:2e-6, and 0.91 for both BioM-ELECTRA-Large-SQuAD2; Lr:6e-6 and BioM-ELECTRA-Large-Discriminator; Lr:6e-6.

**Table 1:** Performance of the Abstract Component Classifier using PubMed dataset.

Model	Learning rate	Epochs	Accuracy	Precision	Recall	*F*_1_-score
BERT^a^	6e-6	4.6	0.89	0.90	0.89	0.89
BioLinkBERT	2e-6	5.8	0.92	0.92	0.92	0.92
BioM-ELECTRA^b^-Large-SQuAD2	6e-6	5.6	0.91	0.91	0.91	0.91
BioM-ELECTRA-Large-Discriminator	6e-6	5.2	0.91	0.91	0.91	0.91

^a^BERT: Bidirectional Encoder Representations from Transformer.

^b^ELECTRA: Efficiently Learning an Encoder that Classifies Token Replacements Accurately.

The performance results by label using the dataset composed of sentences from the abstracts of the articles in the targeted systematic review (Systematic Review Dataset) are shown in Table 2. Implemented classifiers showed the average *F*_1_-scores of 0.88-0.93, 0.93-0.95, 0.92-0.92, and 0.83-0.85 for Introduction, Methods, Results, and Conclusions, respectively. In this study, the BioM-ELECTRA-Large-Discriminator, which achieved a macro–*F*_1_-score of 0.94 in one instance, was selected as the Abstract Component Classifier Model.

**Table 2:** Performance of classifiers implemented by each label using the Systematic Review Dataset.

	BERT^a^			BioLinkBERT				BioM-ELECTRA^b^-Large-SQuAD2				BioM-ELECTRA-Large-Discriminator		
	Precision	Recall	*F*_1_-score	Precision	Recall	*F*_1_-score	Precision		Recall	*F*_1_-score	Precision		Recall	*F*_1_-score
Introduction	0.86	0.89	0.88	0.92	0.94	0.93	0.92		0.92	0.92	0.92		0.93	0.92
Methods	0.94	0.93	0.93	0.96	0.94	0.95	0.92		0.95	0.94	0.94		0.95	0.95
Results	0.92	0.92	0.92	0.93	0.91	0.92	0.92		0.92	0.92	0.95		0.89	0.92
Conclusion	0.84	0.82	0.83	0.81	0.89	0.85	0.87		0.80	0.84	0.80		0.89	0.85
macro average	0.89	0.89	0.89	0.91	0.92	0.91	0.91		0.90	0.90	0.90		0.92	0.91

^a^BERT: Bidirectional Encoder Representations from Transformer.

^b^ELECTRA: Efficiently Learning an Encoder that Classifies Token Replacements Accurately.

##### Development of a Screening Model Using Article Components as Determined by the Abstract Component Classifier Model

Table 3 shows the average results of 5 evaluations of 10 screening models created from the component-composed datasets classified by the developed Abstract Component Classifier Model, along with the results of models created from the same 10 component-composed datasets and all 5 components in Experiment 1. In the tables, among the screening models with the same component composition, those with higher average F10-Scores are highlighted in bold. The results of the screening models using Lr not included in these tables are shown in Table S6-S7 in Multimedia Appendix 1. For the screening model using BERT (Table 3), the model trained on the 4 components Title + Methods + Results + Conclusion recorded the highest F10-Score (precision=0.35; recall=0.93; F10-Score=0.91). The model with the lowest F10-Score that was trained on Title + Methods + Conclusion showed precision=0.40; recall=0.86; and F10-Score=0.85. For BioLinkBERT (Table 4), the model trained on Title + Methods + Results + Conclusion recorded the highest F10-Score (precision=0.42; recall=0.90; F10-Score=0.88). The lowest F10-Score was observed in the model trained on Introduction + Methods (precision=0.63; recall=0.78; F10-Score=0.78). For BioM-ELECTRA (Table 5), the model trained on Methods + Results + Conclusion achieved the highest F10-Score (precision=0.38; recall=0.86; F10-Score=0.85). The lowest F10-Score was obtained for the model trained on Title + Results + Conclusion (precision=0.25; recall=0.69; F10-Score=0.68).

**Table 3:** Comparison of F10-Score between screening models using component data by Abstract Component Classifier Model and screening model using manually classified component data in hand, based on BERT^a^.

	Screening models using component data by the classifier							Screening models using manually classified component data					
Composition	Learning rates	Epochs	Accuracy	Precision	Recall	F10-Score	Learning rates		Epochs	Accuracy	Precision	Recall	F10-Score
T^b^ + M^c^ + R^d^ + C^e^	2e-6	9.2	0.92	0.35	0.93	0.91	2e-6		9.0	0.92	0.35	0.93	0.91
T + I^f^ + R + C	6e-6	6.6	0.92	0.40	0.91	0.89	2e-6		6.8	0.92	0.36	0.91	0.89
T + R	2e-6	8.4	0.88	0.25	0.91	0.89	2e-6		6.8	0.91	0.29	0.89	0.87
T + M + R	6e-7	21.0	0.92	0.34	0.90	0.88	6e-7		18.2	0.91	0.33	0.92	0.90
T + I + R	6e-6	6.4	0.87	0.29	0.91	0.88	2e-6		7.0	0.91	0.30	0.90	0.88
T + I + C	2e-6	8.4	0.93	0.36	0.90	0.88	6e-6		5.0	0.90	0.30	0.92	0.90
T + M	6e-6	6.0	0.86	0.29	0.91	0.88	2e-6		6.0	0.86	0.22	0.92	0.89
T + R + C	6e-6	5.4	0.90	0.28	0.88	0.86	6e-6		6.8	0.89	0.27	0.91	0.89
T + I + M	6e-7	14.6	0.88	0.24	0.88	0.85	2e-6		5.8	0.88	0.26	0.93	0.91
T + M + C	2e-6	12.2	0.94	0.40	0.86	0.85	2e-6		7.6	0.93	0.38	0.89	0.87
T + I + M + R + C	—^g^	—	—	—	—	—	6e-6		5.0	0.83	0.31	0.91	0.87

^a^BERT: Bidirectional Encoder Representations from Transformer.

^b^T: Title.

^c^M: Methods.

^d^R: Results.

^e^C: Conclusion.

^f^I: Introduction.

^g^Not applicable.

**Table 4:** Comparison of F10-Score between screening models using component data by Abstract Component Classifier Model and screening model using manually classified component data in hand, based on BioLinkBERT^a^. Descending order of the F10-Score for screening models using component data by classifier.

Composition	Screening models using component data by the classifier							Screening models using manually classified component data					
	Learning rates	Epochs	Accuracy	Precision	Recall	F10-Score	Learning rates		Epochs	Accuracy	Precision	Recall	F10-Score
T^b^ + M^c^ + R^d^ + C^e^	6e-6	11.2	0.94	0.42	0.90	0.88	6e-6		11.8	0.89	0.29	0.95	0.93
T + M + R	6e-6	10.6	0.94	0.41	0.87	0.86	6e-6		6.4	0.94	0.44	0.91	0.90
T + I^f^ + M	6e-6	6.0	0.95	0.53	0.86	0.85	6e-6		6.4	0.92	0.42	0.91	0.89
T + M	6e-6	5.6	0.90	0.29	0.86	0.84	6e-6		6.4	0.92	0.38	0.89	0.87
I + M + R + C	6e-6	6.2	0.93	0.47	0.85	0.84	6e-6		7.6	0.91	0.30	0.94	0.92
I + R	6e-6	5.2	0.93	0.54	0.84	0.82	6e-6		7.8	0.93	0.43	0.89	0.87
M + C	6e-6	5.6	0.92	0.48	0.84	0.82	6e-6		5.6	0.92	0.43	0.89	0.87
M	6e-6	5.4	0.93	0.35	0.82	0.80	6e-6		6.2	0.91	0.33	0.89	0.87
R + C	6e-6	6.0	0.91	0.29	0.82	0.80	6e-6		6.6	0.90	0.35	0.93	0.90
I + M	6e-6	5.2	0.96	0.63	0.78	0.78	6e-6		7.2	0.90	0.30	0.93	0.91
T + I+ M + R + C	—^g^	—	—	—	—	—	6e-6		7.2	0.95	0.53	0.90	0.89

^a^BERT: Bidirectional Encoder Representations from Transformer.

^b^T: Title.

^c^M: Methods.

^d^R: Results.

^e^C: Conclusion.

^f^I: Introduction.

^g^Not applicable.

**Table 5:** Comparison of F10-Score between screening models using component data by Abstract Component Classifier Model and screening model using manually classified component data in hand, based on BioM-ELECTRA^a^. Descending order of the F10-Score for screening models using component data by classifier.

Composition	Screening models using component data by the classifier							Screening models using manually classified component data					
	Learning rates	Epochs	Accuracy	Precision	Recall	F10-Score	Learning rates		Epochs	Accuracy	Precision	Recall	F10-Score
M^b^ + R^c^ + C^d^	6e-7	17.8	0.94	0.38	0.86	0.85	6e-6		7.4	0.94	0.42	0.87	0.86
M	6e-7	13.2	0.89	0.26	0.86	0.84	6e-6		5.6	0.92	0.38	0.83	0.82
T^e^ + R	2e-6	6.4	0.84	0.19	0.86	0.83	2e-6		5.8	0.88	0.27	0.84	0.82
M + C	6e-6	10.0	0.92	0.38	0.84	0.83	6e-6		7.2	0.93	0.37	0.90	0.88
M + R	2e-6	6.6	0.93	0.38	0.83	0.81	2e-6		9.4	0.93	0.40	0.85	0.83
T + M + C	6e-6	6.8	0.91	0.29	0.82	0.80	6e-6		5.8	0.91	0.40	0.83	0.81
I^f^ + M	6e-6	5.4	0.95	0.53	0.81	0.80	6e-6		6.0	0.92	0.45	0.84	0.82
I + C	2e-6	7.0	0.96	0.59	0.78	0.77	6e-6		6.2	0.94	0.45	0.83	0.82
T + M	6e-6	5.4	0.92	0.32	0.78	0.77	6e-6		5.4	0.92	0.33	0.84	0.83
T + R + C	6e-7	14.0	0.90	0.25	0.69	0.68	6e-6		6.8	0.91	0.39	0.85	0.83
T + I + M + R + C	—^g^	—	—	—	—	—	6e-6		4.8	0.95	0.45	0.76	0.77

^a^ELECTRA: Efficiently Learning an Encoder that Classifies Token Replacements.

^b^M: Methods.

^c^R: Results.

^d^C: Conclusion.

^e^T: Title.

^f^I: Introduction.

^g^Not applicable.

## Discussion

### Principal Results

In this study, we evaluated the significance of selecting components from article titles or abstracts on the performance of screening models for systematic review updates. Our results demonstrated that for all pretrained models, we were able to implement screening models that outperformed those trained on all 5 components. This indicates that the selection of components from the article title or abstracts is beneficial for improving the performance of the screening models. The performance differences between models trained on all components versus selected components may be attributed to the interaction between token limitations and information priority. When using all 5 components, the inclusion of introduction or background sections might have resulted in the loss of crucial information from results or conclusion sections due to the 512-token limit. Furthermore, domain-specific models can show varying performance depending on the input information structure [20]. The 3 pretrained models used in this study showed different component compositions, leading to higher average F10-Scores, despite using the same training datasets. Through our comprehensive exploration of all 31 component combinations, we identified certain combinations that consistently showed superior performance. Specifically, the Title+Method+Result+Conclusion combination demonstrated strong performance across different models, and combinations including Methods and Results components generally performed well. For practical implementations where computational resources are limited, focusing on these high-performing combinations, rather than exhaustively testing all possible combinations, could provide a more efficient path to developing effective screening models while maintaining high performance.

Furthermore, we implemented screening models by using the dataset for each component classified using the developed Abstract Component Classifier Model, which analyzes the semantic content of individual sentences to determine their rhetorical roles, enabling the classification of both structured and unstructured abstracts. Consequently, for the large-uncased model, 7 out of the 10 models outperformed the model trained on all 5 components. For BioM-ELECTRA, 9 out of 10 screening models showed higher performance than the model trained on all 5 components. These results demonstrate that a screening model implemented by combining component classification using a classifier model and component selection can surpass the performance of traditional screening models. However, when using the Abstract Component Classifier in Experiment 2, all 10 screening models using BioLinkBERT demonstrated lower performance than BioLinkBERT’s model trained on all 5 components. This behavior, which differs from our observations in Experiment 1, suggests that BioLinkBERT may be more sensitive to the quality and structure of the component classification. The automatic classification process might disrupt certain patterns or contextual relationships that BioLinkBERT relies on, making it perform better with manually classified components in Experiment 1 but not as well with automatically classified components in Experiment 2. This suggests that the effectiveness of component selection strategies may depend not only on the model’s characteristics but also on how the components are identified and structured. This finding highlights the complex interaction between model architecture, component classification method, and overall performance.

### Comparison With Previous Work

Among the screening models using datasets classified by the developed Abstract Component Classifier, the model trained on Title+Method+Result+Conclusion using BERT achieved the highest average F10-Score (precision=0.35, recall=0.93; F10-Score=0.91). The test set included 40 of the 1000 articles. Therefore, this model reduced 1000 studies that had to be scrutinized manually to an average of 106.3, while extracting an average of 37.2 included articles. Following Cohen (2006) formula for work-saved oversampling, which accounts for both the number of articles to be screened and the model’s recall of 0.93, this represents an 88.6% reduction in screening workload. In a previous study by Qin et al [8], the implemented screening model achieved a sensitivity of 96%, a specificity of 78%, and a 64.1% reduction in workload, thereby demonstrating the rationality and feasibility of using a screening model. The F10-Score of the screening model in Qin’s study exceeded that of the model implemented in this study precision=0.50; recall=0.96; F10-Score=0.95). However, it is important to note that Qin’s study used an ensemble model that integrated 4 different screening models, and the F10-Scores of each individual screening model used to implement the ensemble model were lower than the average F10-Score of the screening model in this study (F10-Scores=0.79, 0.81, 0.91, and 0.81).

In screening models that used the article abstract component classifier, 7 out of 10 models for BERT and 8 models for BioM-ELECTRA underperformed compared to the average F10-Score of models with the same component compositions that were created through manual component classification. For BioLinkBERT, all screening models showed lower average F10-Scores than those of the screening models with manually classified component datasets. These results indicate a tendency for screening models with the classifier model’s classification to underperform when compared with those using manually classified component datasets. This performance gap suggests that our Abstract Component Classifier does not achieve the same level of accuracy as manual labeling, possibly due to limitations in maintaining the contextual integrity of the text during the automatic classification process.

Jiang et al [21] implemented a classifier using the BERT model to classify text into 5 components: background, objective, methods, results, and conclusion and achieved an F1-Score of 0.98 [21]. This F1-Score surpasses that of the classifier implemented in our study. This result may contribute to the differences in the classification methods from previous studies conducted as Machine Reading Comprehension tasks and the scale of the dataset, which used PubMed 200k and comprised approximately 200,000 RCT trial abstracts [22]. However, previous studies used BERTbase13 without a medical literature corpus for pretraining. In our study, the classifiers that used BioLinkBERT, BioM-ELECTRA-Large-SQuAD2, and BioM-ELECTRA-Large-Discriminator achieved higher *F*_1_-scores than the one using BERT. Therefore, it is important to construct a classifier using these pretrained models, and the same method that was used in previous studies could implement a superior classifier.

### Analysis of Model Performance Metrics

In this study, the F10-Score was the primary evaluation metric for the screening model. This choice was made for 2 reasons: to prioritize recall and apply an appropriate penalty to screening models that classify everything as positive. As expected, our results showed that the screening model could be evaluated based on the F10-Score. Moreover, we implemented screening models that had a higher F10-Score than one that classified everything as positive (F10-Score=0.81). This suggests that adopting the F10-Score as the main evaluation metric in our study was appropriate. However, the F10-Score of the screening model classifying everything as positive varied depending on the ratio of the included and excluded articles in the test set. Therefore, it is inferred that when creating test sets with different ratios from those used in our study and conducting similar evaluations, it will be necessary to set a new appropriate β.

Alternative approaches to evaluation metrics, such as maximizing specificity at 99% recall, could also be considered for systematic review screening tasks. While our F10-score approach prioritizes recall while maintaining some consideration for precision, the specificity-focused approach would explicitly maximize the number of excluded articles while maintaining a fixed, very high recall. Both approaches have merit for systematic review updating, though they are optimized for slightly different practical outcomes. Furthermore, F10-scores can be influenced by class prevalence in the test set. In our study, we maintained the class distributions of the test set similar to those of the original systematic review to ensure realistic performance evaluation, reflecting the naturally imbalanced distribution of relevant versus irrelevant articles. Future research might explore how different evaluation metrics and varying class distributions affect model selection and practical utility in systematic review updating, particularly in the context of identifying a small number of relevant articles from a large corpus.

In comparing different models’ performances, we observed that small improvements in the F10-Score often reflected different trade-offs between precision and recall. For example, the BioLinkBERT model trained on Title+Method+Result+Conclusion achieved a higher F10-Score (0.93) compared to BERT (0.91), primarily due to higher recall (0.95 vs 0.93) despite lower precision (0.28 vs 0.35). While this trade-off resulted in a numerically higher F10-Score, it is important to note that in practical applications, significantly lower precision means more irrelevant articles requiring manual screening. However, in the context of systematic reviews, this trade-off might be acceptable given the critical importance of not missing relevant studies, though it suggests the need for careful consideration of the balance between comprehensive coverage and screening efficiency in different systematic review contexts.

### Limitations

One limitation of this study pertains to the generalizability of our results. We implemented and compared the performances of the screening models using the screening results of a single systematic review focusing on adverse events of macrolide antibiotics. While this dataset provided a solid foundation for model development and evaluation, comprehensive validation across multiple systematic reviews in different medical specialties, such as oncology, cardiology, infectious diseases, and pediatrics, is essential to confirm the broad applicability of our approach. Without such validation, the model’s performance may vary significantly across different medical domains due to variations in terminology, study designs, and reporting patterns. For instance, the language and structure used in oncology trial reports may differ substantially from those in used cardiovascular studies, potentially affecting the model’s component classification accuracy and overall screening performance. Our results may not fully represent the model’s performance in systematic reviews of other medical areas (such as cardiovascular disease, and oncology) or different types of studies (such as observational studies or cohort studies). Furthermore, regarding the criteria for classification by component used in this study, it is possible that not all criteria are comprehensively applicable for classifying components in articles from other fields. Another limitation is that our study did not include comparisons with recent large language models (LLMs). Computational resource constraints and reproducibility concerns primarily drove the decision to exclude LLM comparisons, as many current LLMs are not open-source and require significant computational infrastructure for fine-tuning. In addition, the rapid evolution of LLMs poses challenges for establishing stable benchmarks. While we focused on established BERT-based models that are widely validated for classification tasks, recent research has shown that the relative performance of different models can vary significantly based on data availability. For instance, Jahan et al. demonstrated that while LLMs can outperform fine-tuned models on biomedical tasks with limited training data, BERT-based models often maintain advantages when substantial training data is available for fine-tuning [23]. Our current approach using BERT-based models, with access to sufficient training data, provides a solid benchmark for evaluating the effectiveness of component-based classification in systematic review updating.

### Future Work

In this study, we classified each piece of article into 5 components: title and 4 abstract components (introduction, methods, results, and conclusion). Article abstracts can be further subdivided. By refining the component classification, we can improve the performance of the screening model. Therefore, future research that implements screening models using further subdivided articles is required. The dataset used in this study comprised 256 studies. This number is relatively high for the articles analyzed in a systematic review. Our screening model intended to use the included and excluded articles from a preupdated systematic review as the training dataset. Therefore, implementing a screening model for a systematic review that analyzes a small number of studies may result in decreased performance. Therefore, it is necessary to estimate the number of studies required to achieve a performance level comparable to that of the screening model that was developed in this study. Finally, the performance of the screening model was evaluated individually. Therefore, combining the technique of article title or abstract component selection with methods, such as implementing ensemble models, could potentially implement even high-performance screening models.

Future research should also explore strategies to address the few limitations observed in our study. This may include investigating models designed for handling longer sequences, such as Longformer [24] or Big Bird [25], or developing methods to process each abstract component separately before combining their vector representations. While these approaches could improve screening performance, they are likely to increase computational complexity compared to our current method. Therefore, future work will need to explore ways to balance these advanced techniques with computational efficiency requirements.

Investigating the integration of LLMs into systematic review screening presents a promising direction for future research. Consequently, future studies should examine the cost-effectiveness and computational efficiency of various LLM integration strategies, particularly in resource-constrained settings. Research into fine-tuning smaller, more efficient LLMs specifically for systematic review tasks could provide a practical balance between model performance and computational requirements.

### Conclusions

Our study revealed that implementing a screening model trained on selected components of the title or abstract articles could enable better performance than a traditionally implemented model that was trained on all 5 components in some cases. Furthermore, we developed an Abstract Component Classifier that classifies each sentence of the abstract into different components and implements screening models through the component classification of target article abstracts using this classifier and the selection of article title/abstract components. Thus, we were able to implement screening models with a higher performance than the traditional model trained on all 5 components in the 2 pretrained models. This approach could reduce the workload of the systematic review update process.

## Appendix

Multimedia Appendix 1 Additional materials about the details of the search, element composition datasets, and performance of the models.

## Abbreviations

BERT: Bidirectional Encoder Representations from Transformer
ELECTRA: Efficiently Learning an Encoder that Classifies Token Replacements Accurately
LLM: large language model
Lr: learning rates
NLP: natural language processing
WHO: World Health Organization
