# Supplementary material for: Improving Systematic Review Updates With Natural Language Processing Through Abstract Component Classification and Selection: Algorithm Development and Validation
Source: JMIR Med Inform. 2025 Mar 27;13:e65371. doi: 10.2196/65371 (PMC11986382; doi:10.2196/65371)
Supplement: Multimedia Appendix 1 [file medinform_v13i1e65371_app1.docx]

**Table S1. Criteria for each element in Abstract classification**

| Element | Content |
| --- | --- |
| Introduction (Background) | - Problem statement - Example of previous studies - Purpose of the study |
| Method | - Study design - Study site - Designated PICO (Patient & Intervention & Comparison & Outcome) - Study Period - Statistical Analysis |
| Result | - Number of study participants - Background of participants - Endpoint results |
| Conclusion | - Discussion - Limitation - Conclusion |
| Other/Excluded | - Trial number - Funding - Copyright - Element name only - Abstract without any of the above 4 elements (such as Protocol) |

**Table S2. Search terms and formulas.**

| # | Search Term |
| --- | --- |
| 1 | exp Macrolides/ |
| 2 | macrolide*.tw, nm, ot. |
| 3 | (azithromycin* or clarithromycin* or erythromycin* or roxithromycin*).tw, nm, ot. |
| 4 | or/1-3 |
| 5 | exp Placebos/ |
| 6 | placebo*.tw, nm, ot. |
| 7 | 5 or 6 |
| 8 | 4 and 7 |

**Table S3. Element compositions and abbreviations of these used in this study**

|  | Element Composition | Abbreviation |
| --- | --- | --- |
| 1 | Title | T |
| 2 | Introduction | I |
| 3 | Method | M |
| 4 | Results | R |
| 5 | Conclusion | C |
| 6 | Title & | T+I |
| 7 | Title & Method | T+M |
| 8 | Title & Results | T+R |
| 9 | Title & Conclusion | T+C |
| 10 | Introduction & Methods | I+M |
| 11 | Introduction & Results | I+R |
| 12 | Introduction & Conclusion | I+C |
| 13 | Method & Results | M+R |
| 14 | Method & Conclusion | M+C |
| 15 | Results & Conclusion | R+C |
| 16 | Title & Introduction & Methods | T+I+M |
| 17 | Title & Introduction & Results | T+I+R |
| 18 | Title & Introduction & Conclusion | T+I+C |
| 19 | Title & Method & Results | T+M+R |
| 20 | Title & Method & Conclusion | T+M+C |
| 21 | Title & Results & Conclusion | T+R+C |
| 22 | Introduction & Methods & Results | I+M+R |
| 23 | Introduction & Methods & Conclusions | I+M+C |
| 24 | Introduction & Results & Conclusion | I+R+C |
| 25 | Methods & Results & Conclusion | M+R+C |
| 26 | Title & Introduction & Methods & Results | T+I+M+R |
| 27 | Title & Introduction & Methods & Conclusion | T+I+M+C |
| 28 | Title & Introduction & Results & Conclusion | T+I+R+C |
| 29 | Title & Method & Results & Conclusion | T+M+R+C |
| 30 | Introduction & Methods & Results & Conclusion | I+M+R+C |
| 31 | Title & Introduction & Methods & Results & Conclusion | T+I+M+R+C |

**Table S4. Characteristics of datasets used for training and evaluation**

|  | Component | Positive | Negative | Ratio |
| --- | --- | --- | --- | --- |
| Training Set | Title, Introduction, Methods,  Results,Conclusion | 194 | 194 | 1:1 |
| Validation Set | Title, Introduction, Methods,  Results,Conclusion | 22 | 22 | 1:1 |
| Test Set | Title, Introduction, Methods,  Results,Conclusion | 40 | 960 | 1:24 |

Table S5. Performance of the screening models with BERT

| **Element Composition** | **Lr** | **Epochs** | **Accuracy** | **Precision** | **Recall** | **F10-Score** |
| --- | --- | --- | --- | --- | --- | --- |
| T | 6e-7 | 8.8 | 0.80 | 0.15 | 0.86 | 0.82 |
|  | 2e-6 | 5.2 | 0.83 | 0.18 | 0.85 | 0.82 |
|  | 6e-6 | 5.0 | 0.80 | 0.16 | 0.87 | 0.83 |
| I | 6e-7 | 12.8 | 0.92 | 0.31 | 0.83 | 0.81 |
|  | 2e-6 | 6.4 | 0.89 | 0.27 | 0.85 | 0.83 |
|  | 6e-6 | 4.6 | 0.91 | 0.32 | 0.84 | 0.82 |
| M | 6e-7 | 11.2 | 0.91 | 0.30 | 0.83 | 0.81 |
|  | 2e-6 | 5.6 | 0.93 | 0.36 | 0.76 | 0.75 |
|  | 6e-6 | 4.2 | 0.96 | 0.58 | 0.73 | 0.72 |
| R | 6e-7 | 10.4 | 0.91 | 0.28 | 0.81 | 0.79 |
|  | 2e-6 | 5.8 | 0.93 | 0.41 | 0.78 | 0.77 |
|  | 6e-6 | 4.4 | 0.94 | 0.43 | 0.78 | 0.77 |
| C | 6e-7 | 9.4 | 0.94 | 0.39 | 0.72 | 0.71 |
|  | 2e-6 | 5.2 | 0.95 | 0.47 | 0.72 | 0.71 |
|  | 6e-6 | 4.4 | 0.91 | 0.48 | 0.77 | 0.75 |
| T+I | 6e-7 | 10.8 | 0.90 | 0.29 | 0.86 | 0.84 |
|  | 2e-6 | 5.2 | 0.90 | 0.28 | 0.85 | 0.83 |
|  | 6e-6 | 6.0 | 0.86 | 0.24 | 0.88 | 0.85 |
| T+M | 6e-7 | 10.8 | 0.87 | 0.23 | 0.89 | 0.87 |
|  | 2e-6 | 6.0 | 0.86 | 0.22 | 0.92 | 0.89 |
|  | 6e-6 | 6.0 | 0.91 | 0.33 | 0.85 | 0.83 |
| T+R | 6e-7 | 12.4 | 0.89 | 0.26 | 0.88 | 0.86 |
|  | 2e-6 | 6.8 | 0.91 | 0.29 | 0.89 | 0.87 |
|  | 6e-6 | 5.8 | 0.89 | 0.27 | 0.86 | 0.84 |
| T+C | 6e-7 | 11.0 | 0.92 | 0.30 | 0.82 | 0.80 |
|  | 2e-6 | 5.6 | 0.92 | 0.34 | 0.85 | 0.83 |
|  | 6e-6 | 7.4 | 0.92 | 0.36 | 0.86 | 0.84 |
| I+M | 6e-7 | 10.6 | 0.92 | 0.33 | 0.88 | 0.86 |
|  | 2e-6 | 5.6 | 0.91 | 0.32 | 0.88 | 0.86 |
|  | 6e-6 | 5.0 | 0.94 | 0.47 | 0.83 | 0.81 |
| I+R | 6e-7 | 12.0 | 0.94 | 0.44 | 0.82 | 0.81 |
|  | 2e-6 | 6.2 | 0.96 | 0.58 | 0.83 | 0.82 |
|  | 6e-6 | 4.4 | 0.95 | 0.58 | 0.78 | 0.77 |
| I+C | 6e-7 | 9.0 | 0.91 | 0.29 | 0.83 | 0.81 |
|  | 2e-6 | 5.4 | 0.88 | 0.31 | 0.86 | 0.84 |
|  | 6e-6 | 4.2 | 0.93 | 0.51 | 0.80 | 0.79 |
| M+R | 6e-7 | 10.4 | 0.96 | 0.53 | 0.78 | 0.77 |
|  | 2e-6 | 5.4 | 0.97 | 0.67 | 0.77 | 0.77 |
|  | 6e-6 | 4.6 | 0.96 | 0.61 | 0.76 | 0.75 |
| M+C | 6e-7 | 10.8 | 0.93 | 0.37 | 0.82 | 0.80 |
|  | 2e-6 | 10.8 | 0.93 | 0.50 | 0.81 | 0.80 |
|  | 6e-6 | 5.0 | 0.94 | 0.65 | 0.76 | 0.75 |
| R+C | 6e-7 | 14.2 | 0.90 | 0.28 | 0.84 | 0.82 |
|  | 2e-6 | 7.0 | 0.96 | 0.57 | 0.75 | 0.74 |
|  | 6e-6 | 4.0 | 0.88 | 0.59 | 0.78 | 0.77 |
| T+I+M | 6e-7 | 11.4 | 0.89 | 0.28 | 0.92 | 0.90 |
|  | 2e-6 | 5.8 | 0.88 | 0.26 | 0.93 | 0.90 |
|  | 6e-6 | 5.6 | 0.91 | 0.37 | 0.88 | 0.86 |
| T+I+R | 6e-7 | 15.2 | 0.92 | 0.32 | 0.89 | 0.87 |
|  | 2e-6 | 7.0 | 0.91 | 0.30 | 0.89 | 0.88 |
|  | 6e-6 | 5.4 | 0.92 | 0.37 | 0.88 | 0.86 |
| T+I+C | 6e-7 | 10.6 | 0.90 | 0.28 | 0.89 | 0.87 |
|  | 2e-6 | 6.4 | 0.93 | 0.40 | 0.85 | 0.83 |
|  | 6e-6 | 5.0 | 0.90 | 0.30 | 0.92 | 0.90 |
| T+M+R | 6e-7 | 18.2 | 0.91 | 0.32 | 0.92 | 0.90 |
|  | 2e-6 | 8.2 | 0.92 | 0.37 | 0.90 | 0.88 |
|  | 6e-6 | 8.6 | 0.91 | 0.33 | 0.88 | 0.86 |
| T+M+C | 6e-7 | 11.6 | 0.93 | 0.36 | 0.87 | 0.86 |
|  | 2e-6 | 7.6 | 0.93 | 0.38 | 0.89 | 0.87 |
|  | 6e-6 | 5.0 | 0.92 | 0.45 | 0.81 | 0.79 |
| T+R+C | 6e-7 | 11.8 | 0.91 | 0.31 | 0.86 | 0.84 |
|  | 2e-6 | 7.4 | 0.88 | 0.25 | 0.91 | 0.88 |
|  | 6e-6 | 6.8 | 0.89 | 0.27 | 0.91 | 0.89 |
| I+M+R | 6e-7 | 8.8 | 0.94 | 0.48 | 0.79 | 0.78 |
|  | 2e-6 | 6.0 | 0.96 | 0.61 | 0.81 | 0.81 |
|  | 6e-6 | 4.6 | 0.97 | 0.73 | 0.77 | 0.76 |
| I+M+C | 6e-7 | 10.8 | 0.95 | 0.45 | 0.81 | 0.80 |
|  | 2e-6 | 6.8 | 0.95 | 0.55 | 0.79 | 0.78 |
|  | 6e-6 | 4.2 | 0.95 | 0.58 | 0.77 | 0.76 |
| I+R+C | 6e-7 | 14.8 | 0.96 | 0.54 | 0.80 | 0.80 |
|  | 2e-6 | 6.8 | 0.96 | 0.67 | 0.78 | 0.78 |
|  | 6e-6 | 5.8 | 0.95 | 0.56 | 0.81 | 0.80 |
| M+R+C | 6e-7 | 12.4 | 0.96 | 0.59 | 0.78 | 0.78 |
|  | 2e-6 | 8.8 | 0.96 | 0.62 | 0.80 | 0.79 |
|  | 6e-6 | 4.4 | 0.97 | 0.74 | 0.74 | 0.74 |
| T+I+M+R | 6e-7 | 11.8 | 0.89 | 0.30 | 0.88 | 0.85 |
|  | 2e-6 | 6.0 | 0.88 | 0.28 | 0.89 | 0.86 |
|  | 6e-6 | 4.4 | 0.89 | 0.42 | 0.86 | 0.84 |
| T+I+M+C | 6e-7 | 11.4 | 0.95 | 0.45 | 0.84 | 0.83 |
|  | 2e-6 | 6.6 | 0.95 | 0.44 | 0.84 | 0.83 |
|  | 6e-6 | 5.8 | 0.95 | 0.55 | 0.82 | 0.81 |
| T+I+R+C | 6e-7 | 11.8 | 0.94 | 0.39 | 0.84 | 0.82 |
|  | 2e-6 | 6.8 | 0.92 | 0.36 | 0.91 | 0.89 |
|  | 6e-6 | 6.6 | 0.91 | 0.37 | 0.87 | 0.85 |
| T+M+R+C | 6e-7 | 13.6 | 0.93 | 0.36 | 0.87 | 0.85 |
|  | 2e-6 | 9.0 | 0.92 | 0.35 | 0.93 | 0.91 |
|  | 6e-6 | 6.6 | 0.87 | 0.32 | 0.88 | 0.85 |
| I+M+R+C | 6e-7 | 12.6 | 0.95 | 0.51 | 0.79 | 0.78 |
|  | 2e-6 | 6.0 | 0.92 | 0.44 | 0.82 | 0.81 |
|  | 6e-6 | 5.0 | 0.97 | 0.74 | 0.74 | 0.74 |
| T+I+M+R+C | 6e-7 | 11.4 | 0.92 | 0.36 | 0.84 | 0.83 |
|  | 2e-6 | 6.8 | 0.92 | 0.38 | 0.87 | 0.85 |
|  | 6e-6 | 5.0 | 0.83 | 0.31 | 0.91 | 0.87 |

T:Title, I:Introduction, M:Method, R:Result, C:Conclusion

Table S6. Performance of the screening models with BioLinkBERT

| **Element Composition** | **Lr** | **Epochs** | **Accuracy** | **Precision** | **Recall** | **F10-Score** |
| --- | --- | --- | --- | --- | --- | --- |
| T | 6e-7 | 11.4 | 0.85 | 0.16 | 0.64 | 0.62 |
|  | 2e-6 | 6.2 | 0.81 | 0.16 | 0.79 | 0.76 |
|  | 6e-6 | 5.4 | 0.86 | 0.18 | 0.71 | 0.69 |
| I | 6e-7 | 9.2 | 0.98 | 0.85 | 0.73 | 0.73 |
|  | 2e-6 | 5.8 | 0.96 | 0.67 | 0.76 | 0.75 |
|  | 6e-6 | 6.0 | 0.89 | 0.38 | 0.87 | 0.85 |
| M | 6e-7 | 10.0 | 0.97 | 0.59 | 0.71 | 0.71 |
|  | 2e-6 | 5.0 | 0.94 | 0.47 | 0.73 | 0.72 |
|  | 6e-6 | 6.2 | 0.91 | 0.33 | 0.89 | 0.87 |
| R | 6e-7 | 10.6 | 0.95 | 0.45 | 0.71 | 0.71 |
|  | 2e-6 | 6.2 | 0.95 | 0.42 | 0.75 | 0.74 |
|  | 6e-6 | 6.2 | 0.92 | 0.41 | 0.84 | 0.83 |
| C | 6e-7 | 10.6 | 0.98 | 0.85 | 0.71 | 0.71 |
|  | 2e-6 | 5.6 | 0.98 | 0.76 | 0.69 | 0.69 |
|  | 6e-6 | 5.8 | 0.94 | 0.58 | 0.77 | 0.76 |
| T+I | 6e-7 | 8.0 | 0.97 | 0.64 | 0.75 | 0.74 |
|  | 2e-6 | 4.8 | 0.96 | 0.58 | 0.74 | 0.73 |
|  | 6e-6 | 5.4 | 0.92 | 0.41 | 0.86 | 0.85 |
| T+M | 6e-7 | 10.2 | 0.94 | 0.44 | 0.84 | 0.83 |
|  | 2e-6 | 6.8 | 0.93 | 0.38 | 0.84 | 0.82 |
|  | 6e-6 | 6.4 | 0.92 | 0.38 | 0.89 | 0.87 |
| T+R | 6e-7 | 12.0 | 0.93 | 0.34 | 0.80 | 0.79 |
|  | 2e-6 | 5.4 | 0.91 | 0.34 | 0.80 | 0.78 |
|  | 6e-6 | 6.4 | 0.91 | 0.32 | 0.88 | 0.86 |
| T+C | 6e-7 | 10.8 | 0.94 | 0.39 | 0.76 | 0.75 |
|  | 2e-6 | 5.6 | 0.95 | 0.42 | 0.73 | 0.72 |
|  | 6e-6 | 6.2 | 0.93 | 0.39 | 0.85 | 0.83 |
| I+M | 6e-7 | 9.0 | 0.98 | 0.85 | 0.73 | 0.73 |
|  | 2e-6 | 5.4 | 0.95 | 0.51 | 0.73 | 0.72 |
|  | 6e-6 | 7.2 | 0.90 | 0.30 | 0.93 | 0.90 |
| I+R | 6e-7 | 10.0 | 0.98 | 0.86 | 0.73 | 0.73 |
|  | 2e-6 | 5.4 | 0.95 | 0.48 | 0.74 | 0.73 |
|  | 6e-6 | 7.8 | 0.93 | 0.43 | 0.89 | 0.87 |
| I+C | 6e-7 | 7.8 | 0.98 | 0.86 | 0.70 | 0.70 |
|  | 2e-6 | 4.8 | 0.97 | 0.81 | 0.72 | 0.71 |
|  | 6e-6 | 6.0 | 0.94 | 0.54 | 0.82 | 0.81 |
| M+R | 6e-7 | 9.8 | 0.96 | 0.61 | 0.75 | 0.74 |
|  | 2e-6 | 6.0 | 0.95 | 0.46 | 0.74 | 0.73 |
|  | 6e-6 | 5.2 | 0.95 | 0.63 | 0.83 | 0.82 |
| M+C | 6e-7 | 9.0 | 0.98 | 0.87 | 0.71 | 0.71 |
|  | 2e-6 | 5.8 | 0.95 | 0.51 | 0.75 | 0.74 |
|  | 6e-6 | 5.6 | 0.92 | 0.42 | 0.89 | 0.87 |
| R+C | 6e-7 | 10.8 | 0.97 | 0.71 | 0.72 | 0.71 |
|  | 2e-6 | 7.2 | 0.96 | 0.58 | 0.73 | 0.73 |
|  | 6e-6 | 6.6 | 0.90 | 0.35 | 0.93 | 0.90 |
| T+I+M | 6e-7 | 10.6 | 0.98 | 0.86 | 0.73 | 0.73 |
|  | 2e-6 | 5.0 | 0.97 | 0.69 | 0.68 | 0.68 |
|  | 6e-6 | 6.4 | 0.92 | 0.42 | 0.90 | 0.89 |
| T+I+R | 6e-7 | 10.8 | 0.98 | 0.70 | 0.74 | 0.73 |
|  | 2e-6 | 6.2 | 0.93 | 0.38 | 0.77 | 0.76 |
|  | 6e-6 | 6.6 | 0.93 | 0.45 | 0.88 | 0.86 |
| T+I+C | 6e-7 | 8.4 | 0.98 | 0.77 | 0.70 | 0.70 |
|  | 2e-6 | 5.2 | 0.95 | 0.49 | 0.75 | 0.74 |
|  | 6e-6 | 5.4 | 0.95 | 0.49 | 0.80 | 0.79 |
| T+M+R | 6e-7 | 12.6 | 0.97 | 0.70 | 0.76 | 0.75 |
|  | 2e-6 | 7.4 | 0.97 | 0.66 | 0.78 | 0.78 |
|  | 6e-6 | 6.4 | 0.94 | 0.44 | 0.91 | 0.90 |
| T+M+C | 6e-7 | 10.6 | 0.98 | 0.83 | 0.70 | 0.70 |
|  | 2e-6 | 5.6 | 0.93 | 0.46 | 0.75 | 0.74 |
|  | 6e-6 | 6.2 | 0.96 | 0.59 | 0.84 | 0.83 |
| T+R+C | 6e-7 | 11.8 | 0.98 | 0.76 | 0.72 | 0.72 |
|  | 2e-6 | 6.0 | 0.96 | 0.56 | 0.77 | 0.76 |
|  | 6e-6 | 5.8 | 0.92 | 0.41 | 0.87 | 0.85 |
| I+M+R | 6e-7 | 11.0 | 0.98 | 0.87 | 0.72 | 0.72 |
|  | 2e-6 | 6.0 | 0.95 | 0.52 | 0.73 | 0.72 |
|  | 6e-6 | 6.0 | 0.92 | 0.41 | 0.88 | 0.86 |
| I+M+C | 6e-7 | 9.8 | 0.99 | 0.92 | 0.71 | 0.71 |
|  | 2e-6 | 5.8 | 0.97 | 0.74 | 0.74 | 0.73 |
|  | 6e-6 | 6.6 | 0.94 | 0.49 | 0.84 | 0.83 |
| I+R+C | 6e-7 | 9.4 | 0.98 | 0.83 | 0.68 | 0.69 |
|  | 2e-6 | 5.6 | 0.96 | 0.66 | 0.72 | 0.72 |
|  | 6e-6 | 6.4 | 0.93 | 0.49 | 0.86 | 0.85 |
| M+R+C | 6e-7 | 10.6 | 0.98 | 0.79 | 0.73 | 0.73 |
|  | 2e-6 | 6.4 | 0.97 | 0.67 | 0.76 | 0.76 |
|  | 6e-6 | 5.4 | 0.96 | 0.66 | 0.79 | 0.79 |
| T+I+M+R | 6e-7 | 9.8 | 0.98 | 0.89 | 0.72 | 0.72 |
|  | 2e-6 | 5.4 | 0.94 | 0.50 | 0.75 | 0.74 |
|  | 6e-6 | 5.0 | 0.96 | 0.65 | 0.83 | 0.82 |
| T+I+M+C | 6e-7 | 10.0 | 0.99 | 0.95 | 0.71 | 0.71 |
|  | 2e-6 | 5.6 | 0.97 | 0.75 | 0.73 | 0.72 |
|  | 6e-6 | 7.0 | 0.95 | 0.51 | 0.87 | 0.86 |
| T+I+R+C | 6e-7 | 9.8 | 0.98 | 0.79 | 0.71 | 0.71 |
|  | 2e-6 | 6.0 | 0.93 | 0.56 | 0.77 | 0.75 |
|  | 6e-6 | 7.4 | 0.94 | 0.47 | 0.88 | 0.86 |
| T+M+R+C | 6e-7 | 11.2 | 0.98 | 0.75 | 0.72 | 0.72 |
|  | 2e-6 | 8.2 | 0.94 | 0.51 | 0.80 | 0.79 |
|  | 6e-6 | 11.8 | 0.89 | 0.28 | 0.95 | 0.93 |
| I+M+R+C | 6e-7 | 9.4 | 0.99 | 0.92 | 0.71 | 0.71 |
|  | 2e-6 | 6.6 | 0.96 | 0.51 | 0.75 | 0.75 |
|  | 6e-6 | 7.6 | 0.91 | 0.30 | 0.93 | 0.91 |
| T+I+M+R+C | 6e-7 | 10.4 | 0.98 | 0.86 | 0.72 | 0.72 |
|  | 2e-6 | 6.2 | 0.95 | 0.61 | 0.76 | 0.75 |
|  | 6e-6 | 7.2 | 0.95 | 0.53 | 0.89 | 0.89 |

T:Title, I:Introduction, M:Method, R:Result, C:Conclusion

Table S7. Performance of the screening models with BioM-ELECTRA

| **Element Composition** | **Lr** | **Epochs** | **Accuracy** | **Precision** | **Recall** | **F10-Score** |
| --- | --- | --- | --- | --- | --- | --- |
| T | 6e-7 | 11.8 | 0.82 | 0.16 | 0.78 | 0.75 |
|  | 2e-6 | 6.2 | 0.82 | 0.16 | 0.78 | 0.75 |
|  | 6e-6 | 4.2 | 0.82 | 0.17 | 0.83 | 0.79 |
| I | 6e-7 | 11.4 | 0.96 | 0.61 | 0.77 | 0.76 |
|  | 2e-6 | 6.6 | 0.96 | 0.55 | 0.80 | 0.79 |
|  | 6e-6 | 4.8 | 0.93 | 0.45 | 0.82 | 0.80 |
| M | 6e-7 | 12.8 | 0.93 | 0.43 | 0.82 | 0.81 |
|  | 2e-6 | 5.0 | 0.96 | 0.53 | 0.77 | 0.77 |
|  | 6e-6 | 5.6 | 0.92 | 0.38 | 0.83 | 0.82 |
| R | 6e-7 | 9.4 | 0.95 | 0.45 | 0.76 | 0.75 |
|  | 2e-6 | 5.0 | 0.96 | 0.47 | 0.75 | 0.74 |
|  | 6e-6 | 4.0 | 0.96 | 0.48 | 0.75 | 0.74 |
| C | 6e-7 | 10.4 | 0.92 | 0.33 | 0.81 | 0.80 |
|  | 2e-6 | 5.2 | 0.93 | 0.44 | 0.77 | 0.76 |
|  | 6e-6 | 4.4 | 0.94 | 0.56 | 0.75 | 0.74 |
| T+I | 6e-7 | 11.8 | 0.85 | 0.18 | 0.71 | 0.68 |
|  | 2e-6 | 5.8 | 0.91 | 0.28 | 0.68 | 0.67 |
|  | 6e-6 | 4.8 | 0.92 | 0.32 | 0.81 | 0.79 |
| T+M | 6e-7 | 13.8 | 0.88 | 0.21 | 0.72 | 0.70 |
|  | 2e-6 | 6.6 | 0.91 | 0.30 | 0.74 | 0.72 |
|  | 6e-6 | 5.4 | 0.92 | 0.33 | 0.84 | 0.83 |
| T+R | 6e-7 | 13.2 | 0.90 | 0.26 | 0.82 | 0.80 |
|  | 2e-6 | 5.8 | 0.88 | 0.26 | 0.84 | 0.82 |
|  | 6e-6 | 4.4 | 0.89 | 0.26 | 0.78 | 0.76 |
| T+C | 6e-7 | 12.6 | 0.92 | 0.29 | 0.75 | 0.74 |
|  | 2e-6 | 6.2 | 0.92 | 0.33 | 0.75 | 0.74 |
|  | 6e-6 | 5.4 | 0.96 | 0.51 | 0.71 | 0.71 |
| I+M | 6e-7 | 12.0 | 0.96 | 0.65 | 0.77 | 0.77 |
|  | 2e-6 | 7.0 | 0.97 | 0.68 | 0.77 | 0.77 |
|  | 6e-6 | 6.0 | 0.92 | 0.45 | 0.84 | 0.82 |
| I+R | 6e-7 | 11.2 | 0.96 | 0.58 | 0.76 | 0.75 |
|  | 2e-6 | 6.6 | 0.96 | 0.61 | 0.77 | 0.77 |
|  | 6e-6 | 7.0 | 0.96 | 0.63 | 0.80 | 0.79 |
| I+C | 6e-7 | 10.6 | 0.97 | 0.61 | 0.76 | 0.75 |
|  | 2e-6 | 7.4 | 0.97 | 0.65 | 0.76 | 0.75 |
|  | 6e-6 | 6.2 | 0.94 | 0.45 | 0.83 | 0.82 |
| M+R | 6e-7 | 14.4 | 0.93 | 0.38 | 0.84 | 0.82 |
|  | 2e-6 | 9.4 | 0.93 | 0.40 | 0.85 | 0.83 |
|  | 6e-6 | 6.6 | 0.95 | 0.57 | 0.83 | 0.82 |
| M+C | 6e-7 | 15.0 | 0.96 | 0.58 | 0.79 | 0.79 |
|  | 2e-6 | 6.2 | 0.97 | 0.63 | 0.76 | 0.76 |
|  | 6e-6 | 7.2 | 0.93 | 0.37 | 0.89 | 0.88 |
| R+C | 6e-7 | 13.4 | 0.97 | 0.73 | 0.74 | 0.74 |
|  | 2e-6 | 6.8 | 0.97 | 0.80 | 0.74 | 0.73 |
|  | 6e-6 | 10.0 | 0.96 | 0.70 | 0.74 | 0.73 |
| T+I+M | 6e-7 | 10.6 | 0.89 | 0.23 | 0.70 | 0.68 |
|  | 2e-6 | 6.0 | 0.91 | 0.32 | 0.74 | 0.73 |
|  | 6e-6 | 4.8 | 0.93 | 0.37 | 0.73 | 0.72 |
| T+I+R | 6e-7 | 10.8 | 0.90 | 0.26 | 0.74 | 0.73 |
|  | 2e-6 | 5.6 | 0.91 | 0.35 | 0.71 | 0.69 |
|  | 6e-6 | 4.8 | 0.95 | 0.49 | 0.74 | 0.73 |
| T+I+C | 6e-7 | 11.2 | 0.92 | 0.29 | 0.75 | 0.74 |
|  | 2e-6 | 5.6 | 0.92 | 0.33 | 0.74 | 0.73 |
|  | 6e-6 | 4.2 | 0.97 | 0.64 | 0.68 | 0.68 |
| T+M+R | 6e-7 | 12.4 | 0.91 | 0.27 | 0.67 | 0.66 |
|  | 2e-6 | 6.4 | 0.93 | 0.37 | 0.72 | 0.71 |
|  | 6e-6 | 4.4 | 0.94 | 0.58 | 0.74 | 0.73 |
| T+M+C | 6e-7 | 11.8 | 0.92 | 0.30 | 0.65 | 0.64 |
|  | 2e-6 | 6.0 | 0.94 | 0.39 | 0.71 | 0.70 |
|  | 6e-6 | 5.8 | 0.91 | 0.39 | 0.83 | 0.81 |
| T+R+C | 6e-7 | 12.0 | 0.92 | 0.32 | 0.64 | 0.63 |
|  | 2e-6 | 5.8 | 0.94 | 0.35 | 0.65 | 0.64 |
|  | 6e-6 | 6.8 | 0.91 | 0.39 | 0.85 | 0.83 |
| I+M+R | 6e-7 | 14.0 | 0.95 | 0.50 | 0.77 | 0.76 |
|  | 2e-6 | 5.8 | 0.97 | 0.68 | 0.74 | 0.74 |
|  | 6e-6 | 6.0 | 0.97 | 0.67 | 0.79 | 0.79 |
| I+M+C | 6e-7 | 14.2 | 0.97 | 0.63 | 0.77 | 0.76 |
|  | 2e-6 | 10.6 | 0.97 | 0.77 | 0.71 | 0.71 |
|  | 6e-6 | 5.8 | 0.97 | 0.66 | 0.76 | 0.76 |
| I+R+C | 6e-7 | 11.2 | 0.95 | 0.43 | 0.77 | 0.76 |
|  | 2e-6 | 6.0 | 0.94 | 0.46 | 0.78 | 0.78 |
|  | 6e-6 | 7.2 | 0.95 | 0.48 | 0.82 | 0.81 |
| M+R+C | 6e-7 | 14.2 | 0.93 | 0.37 | 0.84 | 0.82 |
|  | 2e-6 | 8.4 | 0.94 | 0.42 | 0.87 | 0.85 |
|  | 6e-6 | 7.4 | 0.94 | 0.41 | 0.87 | 0.86 |
| T+I+M+R | 6e-7 | 10.8 | 0.91 | 0.28 | 0.68 | 0.66 |
|  | 2e-6 | 5.6 | 0.93 | 0.35 | 0.67 | 0.66 |
|  | 6e-6 | 5.4 | 0.95 | 0.47 | 0.78 | 0.77 |
| T+I+M+C | 6e-7 | 11.4 | 0.92 | 0.29 | 0.68 | 0.68 |
|  | 2e-6 | 5.4 | 0.90 | 0.29 | 0.72 | 0.71 |
|  | 6e-6 | 5.4 | 0.94 | 0.48 | 0.75 | 0.74 |
| T+I+R+C | 6e-7 | 11.4 | 0.91 | 0.28 | 0.70 | 0.69 |
|  | 2e-6 | 5.8 | 0.92 | 0.33 | 0.72 | 0.70 |
|  | 6e-6 | 5.0 | 0.87 | 0.24 | 0.81 | 0.79 |
| T+M+R+C | 6e-7 | 11.0 | 0.90 | 0.24 | 0.68 | 0.67 |
|  | 2e-6 | 6.2 | 0.87 | 0.21 | 0.81 | 0.79 |
|  | 6e-6 | 4.4 | 0.93 | 0.46 | 0.74 | 0.73 |
| I+M+R+C | 6e-7 | 13.2 | 0.97 | 0.64 | 0.76 | 0.75 |
|  | 2e-6 | 7.6 | 0.96 | 0.68 | 0.63 | 0.62 |
|  | 6e-6 | 5.2 | 0.96 | 0.65 | 0.78 | 0.77 |
| T+I+M+R+C | 6e-7 | 10.6 | 0.89 | 0.23 | 0.71 | 0.69 |
|  | 2e-6 | 5.8 | 0.89 | 0.24 | 0.74 | 0.72 |
|  | 6e-6 | 4.8 | 0.90 | 0.36 | 0.78 | 0.77 |

T:Title, I:Introduction, M:Method, R:Result, C:Conclusion
